# Summer Stress Mitigation in Rainfed Olive Trees Across Multiple Sites: Comparative Effects on Yield and Oil Quality of Glycine Betaine, Kaolin, and Calcium Carbonate in “Koroneiki” and “Lianolia Kerkyras” Cultivars

**DOI:** 10.3390/plants15091294

**Published:** 2026-04-22

**Authors:** Petros Anargyrou Roussos, Asimina-Georgia Karyda, Chrysa Kotsi, Themistoklis Damianakos, Dionissios Spanos, Panagiota G. Kosmadaki, Maria Zoti

**Affiliations:** 1 Laboratory of Pomology, Department of Crop Science, Agricultural University of Athens, Iera Odos 75, 11855 Athens, Greece; romina_karyda@hotmail.com (A.-G.K.); p1162207@aua.gr (C.K.); t04damianakos@gmail.com (T.D.); dionisis1995@hotmail.gr (D.S.); p1162205@aua.gr (P.G.K.); 2 General Directory of Agriculture, Ministry of Rural Development and Food, 10176 Athens, Greece; mzoti@minagric.gr

**Keywords:** antioxidant capacity, free acidity, free fatty acids, peroxides, phenolic compounds

## Abstract

Olive tree (*Olea europaea* L.) is a major Mediterranean crop, valued for both fruit yield and high-quality oil, yet extreme summer stress, including high temperature, intense irradiance, and water limitation, can substantially reduce productivity and affect oil composition. The objective of the present study was to evaluate the mitigating efficacy of foliar applications of glycine betaine (GB), kaolin (K), and calcium carbonate (CC) under rainfed conditions across three Greek sites on “Koroneiki” (in two sites) and “Lianolia Kerkyras” (in one site) cultivars. Treatments were applied during the summer, and effects on fruit yield, oil content per fruit, oil yield per tree, and key oil quality parameters—including total phenols, flavonoids, antioxidant capacity, and fatty acid composition—were assessed. GB significantly enhanced fruit yield and oil production for “Koroneiki” at the site with the harshest environmental conditions (24.37 kg fruits per tree and 4.69 kg of oil per tree compared to 19.16 kg fruits per tree and 3.48 kg of oil per tree in control). In contrast, K proved most effective at the other two sites for both cultivars (43% and 52.8% increase in fruit yield and oil mass per tree in “Koroneiki” respectively and 30% as well as 34% increase in yield and oil mass per tree in “Lianolia Kerkyras”, respectively. CC exhibited limited impact on both productivity and quality. Under all treatments, the oils produced could be classified as extra virgin olive oils, with the products exhibiting minor effects on the functional properties of the oils. These findings indicate that the efficacy of stress-alleviating foliar treatments is strongly influenced by both environmental conditions and cultivar. Overall, K was the most effective treatment, followed by GB. Tailored application of these treatments represents a sustainable approach to maintaining olive productivity and preserving oil quality in the context of climate change.

## 1. Introduction

The olive tree (*Olea europaea* L.) is one of the most ancient and emblematic crops of the Mediterranean Basin, with a cultivation history spanning over 7000 years [1]. It represents a vital socioeconomic and environmental pillar of the region, serving as a primary source of employment and a fundamental component of the rural landscape [2]. Beyond its profound cultural and mythological significance, particularly in Greece, the olive tree is a key agricultural species primarily grown for oil production. As a sclerophyllous species, it is well-adapted to the semi-arid conditions of the Mediterranean agroecosystems [3].

The exceptional quality of olive oil, particularly its health-promoting properties, is largely defined by its phenolic, tocopherol and sterol content, which collectively define its antioxidant capacity and oxidative stability over time [4,5,6]. However, both the qualitative and quantitative profiles of these bioactive compounds result from complex interactions among several factors [6,7]. According to the literature, the primary determinants include the genotype (cultivar), the fruit ripening stage at harvest, and the extraction technology employed [8,9]. In addition, climatic and agronomic conditions, together with soil factors, play a fundamental role in shaping the oil’s chemical profile. Climatic variables—particularly temperature and water availability during fruit set, growth, and oil accumulation—act as major stressors that can either stimulate or inhibit the synthesis of secondary metabolites [9]. The interaction between environmental constraints and the plant’s physiological responses ultimately dictates the nutritional and organoleptic characteristics of the final product, underscoring the importance of implementing protective measures against extreme summer conditions to safeguard olive oil quality [2].

Despite its inherent resilience, the olive tree is increasingly subjected to severe summer stresses, characterized by the synergistic effects of drought, extreme temperatures, and high irradiance [2]. These environmental constraints, which are predicted to increase in frequency due to climate change [10], have negative repercussions on water relations, carbon assimilation, and nutrient uptake, directly affecting its growth and productivity potential. Consequently, these combined stressors threaten the economic viability of the olive sector, making the implementation of water-saving strategies and mitigation measures crucial for its future sustainability [2].

Plants have evolved a wide array of adaptive defense mechanisms to cope with abiotic stress. Among these is the rapid accumulation of compatible solutes, known as osmolytes, including proline, sugars, polyols, and notably glycine betaine (GB) [11,12,13]. These low-molecular-weight organic metabolites can be accumulated at high concentrations without disrupting intracellular biochemistry, playing a vital role in the protection of cell membranes and proteins [14]. Glycine betaine (N,N,N-trimethylglycine), an amphoteric quaternary amine, is primarily synthesized in chloroplasts, where it protects thylakoid membranes, maintains photosynthetic efficiency, and preserves the functional integrity of photosystem II (PSII) [11,12,15]. Beyond its osmoregulatory function, GB alleviates oxidative stress by enhancing the activity of antioxidant enzymes and stabilizing protein structures [11,13,16]. As the endogenous accumulation of GB in many plant species—including olive—is often insufficient to counteract severe environmental stress, its exogenous application via foliar spraying has emerged as an effective and sustainable agronomic practice [11,16]. Foliar-applied GB is readily absorbed by leaf tissues and has been shown to improve stomatal conductance, enhance net photosynthesis (Pn), and promote vegetative growth, offering a cost-effective strategy for mitigating stress and sustaining crop productivity under climate change conditions [14].

Beyond the application of osmolytes, an increasingly prominent strategy for the simultaneous mitigation of multiple abiotic stresses is the implementation of particle film technology (PFT) [2,13]. This innovative approach is based on coating plant surfaces with a protective film of particles. The major representative in this category is kaolin clay particles (K) (an inert aluminosilicate mineral, Al_4_Si_4_O_10_(OH)_8_), specifically formulated to shield crops from the synergistic effects of high temperature, excessive solar irradiance, and water deficit [17]. Unlike traditional methods, kaolin acts as a physical barrier reflecting harmful ultraviolet (UV) and infrared (IR) radiation while allowing the transmission of essential photosynthetically active radiation (PAR) [17]. This reflective property leads to a substantial reduction in leaf and fruit temperatures—often by 3 °C to 6 °C—thereby alleviating heat stress and preventing solar injury or chlorophyll degradation [2,3,13,17,18].

By cooling the canopy, kaolin serves as effective anti-transpirant, enabling stomata to remain functional under moderate stress conditions and improving leaf gas exchange and water-use efficiency [3,18]. Furthermore, while it was originally developed for pest suppression, such as the control of the olive fruit fly (*Bactrocera oleae Rossi*), kaolin has since demonstrated multifunctional benefits, including enhanced vegetative growth, fruit quality, and yield, making it a sustainable and cost-effective tool for maintaining olive productivity under increasingly harsh Mediterranean summer conditions [18,19,20].

A further advancement in particle film technology involves the foliar application of calcium carbonate (CaCO_3_) (CC). Similar to kaolin, calcium carbonate forms a non-toxic reflective barrier that reduces the thermal load on leaves and fruits by reflecting UV and IR radiation without limiting photosynthesis [21]. Beyond its reflective properties, which have been proven effective in reducing sunburn in crops like pineapple, kinnow mandarin and tomato [21,22,23], CaCO_3_ also serves as a source of calcium, a vital nutrient for maintaining fruit quality and structural integrity [24]. Calcium supplementation enhances cell wall strength through the formation of calcium pectate and contributes to the maintenance of membrane integrity, delays senescence processes, and promotes the accumulation of secondary metabolites such as phenolic compounds and ascorbic acid [22,23,25].

Although GB and K have been extensively evaluated in various crops, including olive, under different stress conditions, CC remains comparatively understudied. Therefore, the aim of the present study was to evaluate the effects of three commercially available products containing glycine betaine, kaolin, or calcium carbonate on olive trees grown under rainfed conditions at three distinct locations in Greece. Treatment efficacy was assessed based on fruit yield, olive oil content, oil yield per tree, basic olive oil quality parameters, and functional properties of the extracted olive oil.

## 2. Results

### 2.1. Efficacy Evaluation of the Various Products on Yield, Fruit and Shoot Characteristics

The application of GB under the conditions of Trial 1 (“Koroneiki” cultivar in trial site 1) resulted in the highest fruit yield per tree, reaching 24.37 kg of olives, followed by K, with no significant difference between the two treatments (Table 1). Control trees exhibited yields comparable to those of Kaolin-treated trees and did not differ significantly from trees treated with CC. Under the conditions of Trial 2 (“Koroneiki” cultivar in trial site 2), the highest olive yield was recorded in trees treated with K, followed by those treated with GB, without any significant difference (Table 1). Control trees exhibited the lowest yield, without a difference from CC-treated trees. In “Lianolia Kerkyras” Kaolin-treated trees produced the highest yield among treatments (Table 2).

No significant differences among treatments were observed regarding oil content per fruit and maturity index in all cultivars (Table 1 and Table 2), nor regarding mean shoot length (in “Koroneiki” cultivar) or fruit weight, length and diameter in “Lianolia Kerkyras”.

Nevertheless, the oil yield per tree was significantly higher in GB-treated trees in “Koroneiki” in trial site 1 (Table 1), while Kaolin-treated trees produced the highest oil yield per tree in both trial sites 2 (“Koroneiki”) (Table 1) and 3 (“Lianolia Kerkyras”) (Table 2).

### 2.2. Efficacy Evaluation of the Various Products on Olive Oil Quality Characteristics

Significant differences regarding oil acidity were determined only in the oils produced in trial site 1 (Table 2), where the lowest free oil acidity was determined in the oil produced from trees treated with K, followed by that from trees treated with CC (Table 3). There were not any differences among treatments regarding the peroxide concentration and the UV absorbance indexes (K232 and K270) in any of the oils produced in the three trial sites.

### 2.3. Efficacy Evaluation of the Various Products on Olive Oil Phenolic Compounds and Antioxidant Capacity

The highest total phenol concentration was determined in the oils produced from trees treated with GB during Trial 1 in “Koroneiki” cultivar, followed by control and K (Table 4). In contrast, oils from CC-treated trees exhibited the lowest total phenolic content. On the other hand, the highest total phenol concentration in Trial 2 was determined in oils produced from control trees, followed by those oils produced from Kaolin-treated trees. On the other hand, though, the lowest total phenol concentration in “Lianolia Kerkyras” oils was determined in Kaolin-treated trees, while the highest one was determined in CC-treated ones.

The concentration of o-diphenols did not differ in the oils produced from “Koroneiki” trees under all treatments in both trial site 1 and 2 (Table 4). On the other hand, though, Kaolin-treated “Lianolia Kerkyras” trees produced oils with the lowest o-diphenol concentration.

The total flavonoid concentration did not differ in any of the oils produced in trial sites 1 (“Koroneiki” cultivar) and 3 (“Lianolia Kerkyras” cultivar) (Table 4). On the other hand, GB-treated “Koroneiki” trees grown in trial site 2 produced oils with the lowest total flavonoid concentration.

The antioxidant capacity of the oils measured by the FRAP assay did not differ among treatments in any of the trial sites in which the present experiment took place (Table 4). In contrast, though, the antioxidant capacity (measured by the DPPH assay) of the oils produced from the Kaolin-treated “Koroneiki” trees in trial site 2 was the highest among treatments, with the lowest being determined in oils from CC-treated trees.

### 2.4. Efficacy Evaluation of the Various Products on “Lianolia Kerkyras” Olive Oil Individual Phenolic Compounds and α-Tocopherol Concentration (Trial Site 3)

No significant differences were detected among oils produced under the different treatments, regarding the individual phenolic compounds detected (Table 5). Oleocanthal was the most abundant phenolic compound, followed by oleacein, luteolin, tyrosol and hydroxytyrosol. α-tocopherol concentration was highest under GB treatments and lowest under K treatment.

### 2.5. Efficacy Evaluation of the Various Products on “Lianolia Kerkyras” Olive Oil Fatty Acid Methyl Ester Content and Squalene Concentration (Trial Site 3)

Among the FAMEs, only the content of C:20 was significantly affected by the treatments, with the highest concentration determined in oils produced in control treatment (Table 6).

The treatments did not affect the various groups of free fatty acids in the oils produced, while squalene concentration was highest under control treatment and lowest under K treatment (Table 7).

### 2.6. Hierarchical Cluster Analyses of the Raw Data Produced from the Three Trial Sites

**Figure 1:**
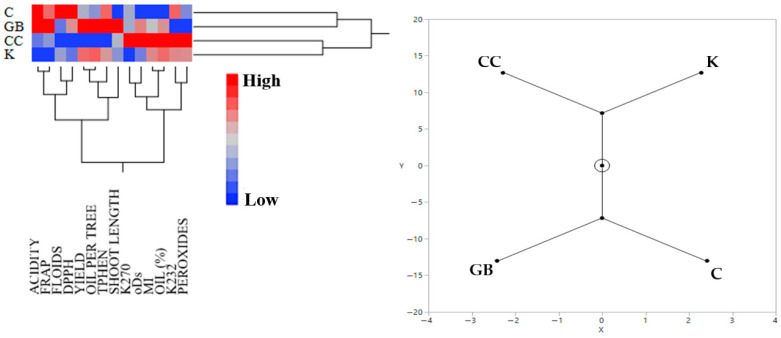
Hierarchical cluster analysis (**left**) and the corresponding constellation plot (on the **right**) of the effects of the various treatments on the measured variables from the data of Trial 1. Abbreviations: ACIDITY, olive oil acidity, FRAP, antioxidant capacity based on ferric reducing antioxidant power assay, FLOIDS, total flavonoids, DPPH, antioxidant capacity based on diphenyl-picryl hydrazyl assay, TPHEN, total phenols, oDs, total o-diphenols, MI, maturity index, OIL (%), oil percentage per mass of fruit, PEROXIDES, olive oil peroxides. K, kaolin, GB, glycine betaine, CC, calcium carbonate, C, control.

Hierarchical cluster analysis of the data of Trial 1 (“Koroneiki” cultivar) revealed a weak association between the control and GB treatments, as well as between the CC and K treatments (Figure 1). The control treatment was characterized by high oil acidity, elevated flavonoid concentration, and increased antioxidant capacity as determined by the DPPH assay. Oils from GB-treated trees were associated with high oil acidity and antioxidant capacity (FRAP), along with increased fruit and oil yield per tree, greater mean shoot length, and elevated total phenolic content, while exhibiting low K232 values and peroxide concentrations. The CC treatment was characterized by high K270 and K232 values, increased o-diphenol and peroxide concentrations, and a high maturity index. Finally, the kaolin treatment was associated with low oil acidity, reduced antioxidant capacity (FRAP), and low K270 values.

**Figure 2:**
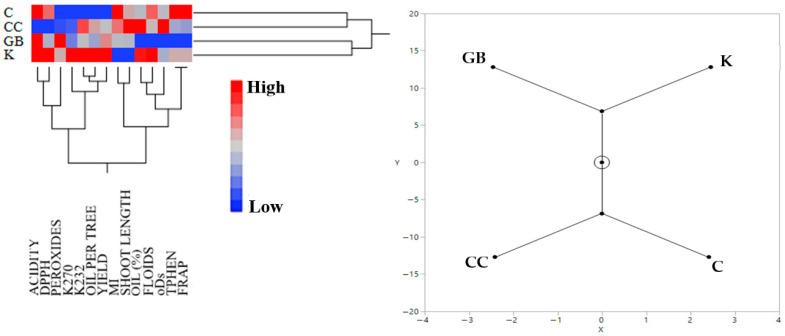
Hierarchical cluster analysis (**left**) and the corresponding constellation plot (on the **right**) of the effects of the various treatments on the measured variables from the data of Trial 2. Abbreviations: ACIDITY, olive oil acidity, FRAP, antioxidant capacity based on ferric reducing antioxidant power assay, FLOIDS, total flavonoids, DPPH, antioxidant capacity based on diphenyl-picryl hydrazyl assay, TPHEN, total phenols, oDs, total o-diphenols, MI, maturity index, OIL (%), oil percentage per mass of fruit, PEROXIDES, olive oil peroxides. K, kaolin, GB, glycine betaine, CC, calcium carbonate, C, control.

The hierarchical clustering analysis of the data from Trial 2 (“Koroneiki” cultivar) revealed a weak relationship between C and CC treatments, as well as between GB and K treatments (Figure 2). The control treatment was associated with oils exhibiting high free acidity, similar to that of GB and K treatments, together with a high fruit maturity index, elevated total phenolic content, and increased antioxidant capacity as determined by the FRAP assay. Oils from CC-treated trees were characterized by low free acidity and reduced antioxidant capacity (DPPH assay), but high o-diphenol concentration and oil content per fruit. The GB treatment was associated with low oil content per fruit, as well as reduced concentrations of total flavonoids, o-diphenols, and total phenols, and low antioxidant capacity measured by the FRAP assay. High fruit yield and oil yield per tree were distinctive characteristics of the K treatment.

**Figure 3:**
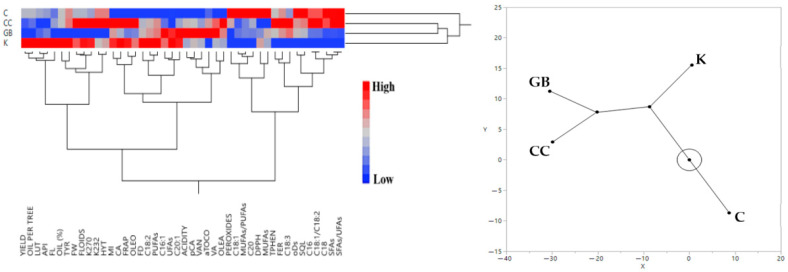
Hierarchical cluster analysis (**left**) and the corresponding constellation plot (on the **right**) of the effects of the various treatments on the measured variables on fruit and olive oil of “Lianolia Kerkyras” cultivar. Abbreviations: ACIDITY, olive oil acidity, FRAP, antioxidant capacity based on ferric reducing antioxidant power assay, FLOIDS, total flavonoids, DPPH, antioxidant capacity based on diphenyl-picryl hydrazyl assay, TPHEN, total phenols, oDs, total o-diphenols, MI, maturity index, OIL (%), oil percentage per mass of fruit, PEROXIDES, olive oil peroxides, FL, fruit length, FW, fruit fresh weight. FD, fruit diameter, VAN, vanillin, CA, caffeic acid, VA, vanillic acid, oDs, total o-diphenols, OLEO, oleocanthal, FER, ferulic acid, LUT, luteolin, API, apigenin, SQl, squalene, TYR, tyrosol, aTOCO, a-tocopherol, pCA, p-coumaric acid, HYT, hydroxytyrosol, OLEA, oleacein, SFAs, saturated fatty acids, UFAs, unsatursated fatty acids, MUFAs, mono-unsaturated fatty acids, PUFAs, poly-unsaturated fatty acids, K, kaolin, GB, glycine betaine, CC, calcium carbonate, C, control.

The hierarchical clustering of the raw data of trial site 3, regarding the efficacy of the various products on “Lianolia Kerkyras” cultivar, revealed distinct groupings (Figure 3). Glycine betaine and CC treatments elicited comparable physiological responses in the olive trees under the study’s environmental conditions, as they clustered closely together. In contrast, the K treatment was positioned on a separate primary branch, significantly distant from the control, and closer to CC and GB treatments. The associated heatmap elucidates this separation, showing that K treatment consistently maintained high values across key performance and quality indicators, such as yield, oil production per tree, and luteolin concentration, while the C group exhibited low values for the same variables.

### 2.7. Discriminant Analyses of the Raw Data Produced from the Three Trial Sites

**Figure 4:**
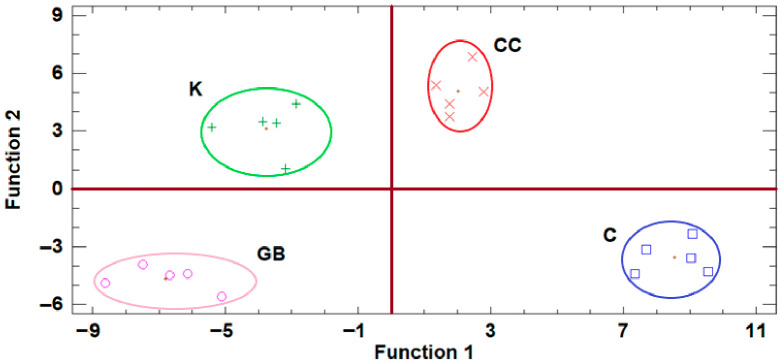
Plot of discriminant functions of the various treatments from the data of Trial 1. K, kaolin, GB, glycine betaine, CC, calcium carbonate, C, control.

The discriminant analysis of the data of Trial 1 in “Koroneiki” cultivar revealed that the effects of the applied treatments were enough to separate them into different quadrants (Figure 4). Control was located in the positive side of Function 1 as CC, but in the negative side of Function 2, opposite to CC. GB was located in the negative sides of both Functions 2 and 1, while K was also found in the negative side of Function 1.

**Figure 5:**
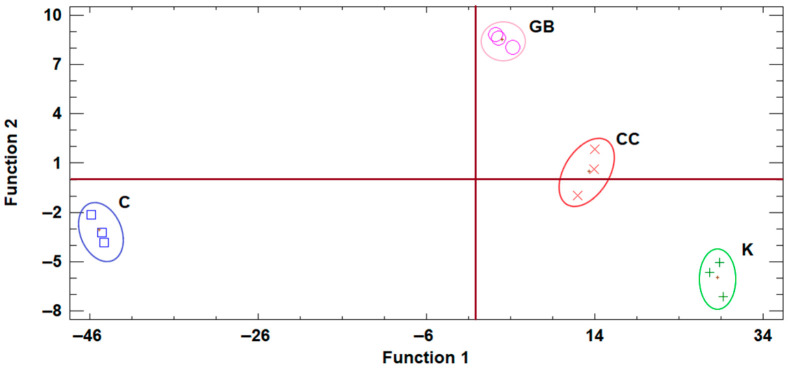
Plot of discriminant functions of the various treatments from the data of Trial 2. K, kaolin, GB, glycine betaine, CC, calcium carbonate, C, control.

The discriminant analysis based on the data from Trial 2 revealed a very interesting result (Figure 5). All treatments involving stress-alleviating products were positioned on the positive side of Function 1, whereas the control treatment was located on the negative side, clearly indicating the overall efficacy of the applied products. Although the three treatments (GB, CC, and K) were positioned in close proximity, their data regions did not overlap, highlighting distinct treatment-specific responses. In particular, K was positioned on the negative side of Function 2, while GB was located on the positive side.

**Figure 6:**
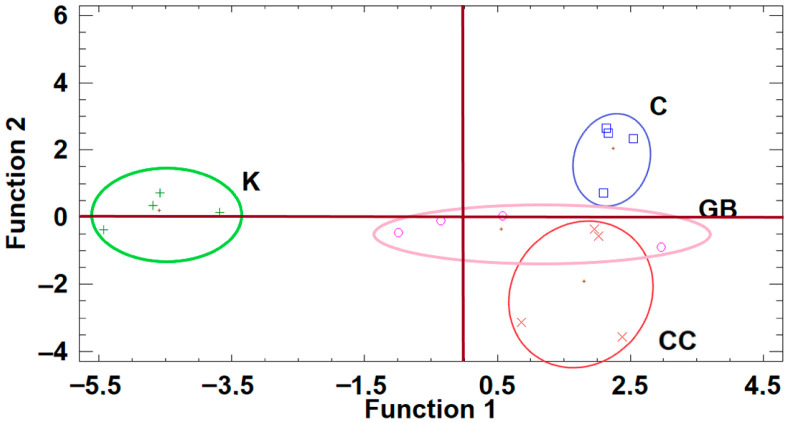
Plot of discriminant functions of the various treatments on “Lianolia Kerkyras” cultivar. K, kaolin, GB, glycine betaine, CC, calcium carbonate, C, control.

Based on the discriminant analysis of the raw data produced from the effects of the various treatments on “Lianolia Kerkyras” cultivar in trial site 3, K treatment was positioned on the negative side of Function 1, far from the other groups (Figure 6). On the other hand, GB and CC treatments showed distinct centroids, but also common areas, reinforcing the clustering results, while control treatment was clearly separated from all other treatments.

## 3. Discussion

In all three trials conducted, both the hierarchical cluster analysis and the discriminant analysis revealed significant differences among treatments, which in most cases were sufficient to fully discriminate them. This led to the robust conclusion that the applied treatments exerted distinct and significant impacts on the measured variables.

The meteorological conditions markedly differed among the three experimental sites. Trial 1 (conducted on “Koroneiki” cultivar) was characterized by extremely high summer temperatures from July to September (frequently exceeding 41 °C), with mean maximum temperatures consistently above 37 °C. Coupled with minimal rainfall, these conditions can be considered harsh, even for the drought-resistant olive tree acclimatized to Mediterranean climates [10,26]. Under these harsh conditions, GB and K demonstrated substantial efficacy, increasing fruit yield by 27% and 18%, respectively, and oil production per tree by 35% and 26%, respectively.

The area where the Trial 2 took place (the cultivar was also “Koroneiki”) experienced a hot summer with mean maximum temperatures approaching 35 °C (peaking at 39 °C) and limited rainfall in August and September. Under these conditions, K proved to be the most effective treatment, increasing fruit yield and oil production per tree by 43% and 52.8%, respectively, followed by GB and CC.

In contrast, the area where Trial 3 on the “Lianolia Kerkyras” cultivar was conducted was characterized by comparatively milder summer conditions. The mean maximum temperature did not exceed 33 °C, with the peak temperature of 37.8 °C recorded in August. September was notably cooler (mean maximum 28.6 °C) and wetter, with substantial rainfall (40 mm in August and 122 mm in September). Under these conditions, K application again significantly enhanced fruit yield (by 30%) and oil production per tree (by 34%).

These results, when considered collectively, demonstrate that both environmental conditions and cultivar strongly modulate the efficacy of the applied products, in agreement with previous reports [3,13,27,28]. Interestingly though, GB seemed to be more efficient than K on increasing yield under the more harsh conditions of Trial 1, as has been previously found [13]. On the other hand, “Koroneiki” trees grown under the milder but still stressful conditions of Trial 2 exhibited higher yield and oil production per tree when treated with K, similar to previous reports [2,27]. It appears that K, acting both as a reflective material—which lowers leaf temperature—and as a water-saving agent which enhances photosynthetic capacity [13], is generally more effective under these conditions for “Koroneiki” than the osmolyte GB, which primarily functions by preserving leaf water content. Calcium carbonate, despite its reflective properties, appeared less effective than K, resulting in lower yield and oil production in both trials. Similar results have been reported by Ghani et al. [29] working with two olive cultivars, i.e., “Aggezi” and “Picual”, while K proved to be superior to CC also in eggplant, sweet orange and apple, based on various yield and quality parameters assessed [30,31,32]. Increases in yield after CC application (using the same product tested here, i.e., Purshade) compared to control have also been reported in two cultivars of date palm, i.e., “Zaghloul” and “Samany” [33], whereas no effects were reported in tomato and sweet pepper [25,34]. In the present study, CC did not consistently enhance olive productivity under rainfed summer conditions, particularly in Trials 1 and 3.

The most informative findings regarding treatment efficacy emerged from Trial 3, where “Lianolia Kerkyras” was used as the experimental cultivar. This cultivar is characterized by larger leaves and higher requirements for soil and atmospheric humidity compared to “Koroneiki”, which explains its traditional cultivation in northwestern Greece [35]. The most favorable results regarding olive fruit and oil production were achieved following the application of K, while CC and GB yielded no significant improvements. Under the cooler and wetter conditions of this site, K was the only treatment that significantly enhanced fruit and oil yield, whereas the effects of GB and CC were negligible. Given the cultivar’s large leaf area and the adequate soil moisture provided by rainfall, osmotic stress was likely minimal; however, excessive solar irradiance and heat load remained critical constraints. Canopy cooling through K application appears to have alleviated heat-induced photosynthetic impairment, thereby explaining the observed yield response. This justifies the improvement in yield and oil production under the influence of K and the limited efficacy of GB, as no significant osmotic stress occurred under these specific environmental conditions.

Nonetheless, GB protective action against heat stress has been reported in a number of other species [11], even though K has been found to be more effective than GB in reducing heat load in various olive cultivars [3,13,28]. A clear gradient in the relative efficacy of K and GB was observed across trials: GB was more effective under the hottest and driest conditions (Trial 1), whereas K dominated under milder but still stressful conditions (Trial 2). In the northernmost trial location—characterized by a cooler, more humid climate and a cultivar less tolerant to drought and heat stress—K remained more effective than GB. However, its relative efficiency declined compared to previous results: the 43% and 53% increases in fruit yield and oil production observed in Trial 2 dropped to 30% and 35%, respectively, in Trial 3. This comparative reduction in efficacy may be attributed to differences in genetic background (comparing “Koroneiki” with “Lianolia Kerkyras”) as well as to the distinct pedoclimatic conditions of the northern site. Previous studies have shown that the effectiveness of K in alleviating heat stress decreases in cooler northern environments [36], particularly when photosynthesis is used as a stress indicator. In the present study, however, mean maximum temperatures in Trial 3 still exceeded the upper threshold for optimal photosynthesis (35 °C) [26], suggesting that K remained beneficial. These findings reinforce the notion that K application rate and frequency must be carefully adjusted to local environmental conditions [36,37].

Overall, the effects of the alleviating products on olive oil quality indices were less pronounced than those on yield. Only in the hottest environment (Trial 1) was oil acidity significantly reduced by treatments, particularly by K, consistent with previous reports [8,19,38]. This reduction is commonly attributed to lower canopy temperatures and improved fruit hydration status [8], as oil acidity is generally lower in cooler and well-irrigated environments [39,40]. The absence of significant acidity differences in Trials 2 and 3 further supports this interpretation. Oils from CC-treated trees in Trial 1 also exhibited low acidity, possibly due to improved fruit moisture retention, as previously reported for the “Picual” olive cultivar [29]. In contrast, GB application did not significantly affect oil acidity in any trial, in agreement with earlier findings in “Memecik” olive cultivar [28].

Peroxide value, K232 and K270 were not significantly affected by treatments across all trials, in agreement with several studies on K application in olive [2,27,36,38], although contrasting results have also been reported [8,19,38], highlighting the complexity of oxidative stability responses. Importantly, the oils produced from all treatments remained within the regulatory limits for EVOO classification.

Phenolic compounds and antioxidant capacity were only moderately influenced by treatments, and their response varied among trials. The lowest phenolic compounds were observed in CC-treated trees in Trial 1, in GB-treated trees in Trial 2, and in Kaolin-treated trees in Trial 3. Generally, an increase in phenolic compounds is expected under stress conditions, as they are one of the many components of plant arsenal against stress [2,41]. According to [42] the effect of CC appears to be species-dependent, potentially mediated through differential regulation of phenylalanine ammonia lyase activity, the key enzyme responsible for the synthesis of phenolic compounds [43]. Under normal conditions, though, or conditions that alleviate stress impact, this enzyme is used to synthesize proteins [41]. Taking this into consideration, it can be assumed that K treatment resulted in low oil phenolic concentration due to the high degree of alleviation it achieved during Trial 3. However, this pattern did not consistently apply across trials, as high yields under GB (Trial 1) and K (Trial 2) were accompanied by elevated phenolics and antioxidant capacity. These findings underline the multifactorial regulation of olive oil phenolic composition. Furthermore, it is known that the concentration of antioxidant molecules, such as phenolic compounds, tocopherols and the antioxidant capacity of the oil, are influenced by multiple factors [8,44]. Among them, the environmental factors prevailing during the growing season play a significant role [8,27,45]. There are reports where contradicting results on the effects of alleviating products on olive oil phenolic compounds have been documented on the same cultivar, during a multi-year experimental period, due to the distinctive environmental conditions that characterized the different seasons [27,36]. Among the various explanations given, the fruit moisture content plays a significant role, since high moisture negatively affects the transferring of phenols to the oil during malaxation [27,46]. The high precipitation levels in Trial 3, combined with the cooler climate and the water-conserving efficacy of K, may partially explain the low phenol concentration in the extracted oil. Specifically, the fruits likely maintained sufficient moisture to suppress the transfer of phenolic compounds to the oil phase during extraction [27,46]. The interplay among environmental factors, agronomic practices, fruit maturity stage, and genetic material creates a multifaceted dynamic that cannot be explained through a univariate approach [47].

The prevalent fatty acids (measured as FAMEs) in the oils produced in Trial 3 were oleic acid, palmitic acid and linoleic acid, in accordance with the literature [2,8,48]. The various treatments did not have a significant effect on the content of free fatty acids, as only arachidic acid (C20:0), a minor fatty acid in olive oil, was significantly reduced. These findings are in contrast to those reported by other researchers, where alleviating products induced an increase in major unsaturated free fatty acids in olive oil [8,48]. Results similar to ours have been reported after K and zeolitite applications on olive cultivar “Correggiolo” [36], as no significant difference concerning the content of the two major fatty acids, i.e., oleic (C18:1) and palmitic acid (C16:0), was observed. This lack of difference among treatments was also corroborated by the free acidity measurements, as it reflects the hydrolytic breakdown of triglycerides into di- and monoglycerides, yielding free fatty acids [2]. This breakdown is accelerated by heat and there are several authors who suggest that increased fruit temperature combined with water shortage leads to increased acidity [39,40]. As the environmental conditions prevailing in Trial 3 were milder than in the other two areas, along with the significant rainfall events that occurred during the later stages of fruit development, it can be assumed that these conditions may have diminished any possible effects of the alleviating products on fatty acid composition. Similarly, squalene concentration did not differ among treatments, as has been previously reported in oils produced from irrigated trees under the influence of various mineral clay particles used as ameliorating products [2], strengthening the assumption that favorable environmental conditions may reduce the efficacy of any alleviation product.

The comparative analysis of the three alleviating treatments highlights distinct differences in their effectiveness under summer stress. Kaolin excelled in both “Koroneiki” (across Trials 1 and 2) and “Lianolia Kerkyras”, primarily through its reflective properties. Kaolin likely reduced canopy temperature and photoinhibition, thereby sustaining both yield and key oil quality attributes, such as phenolic content and antioxidant capacity. Glycine betaine enhanced fruit yield and oil production in Trial 1 in “Koroneiki”, demonstrating that it can positively impact olive production under specific conditions. In contrast, CC showed limited efficacy; its reflective properties appeared insufficient to significantly mitigate heat and irradiance stress. Overall, the results indicate that K is superior to the other treatments under Greek summer conditions, whereas GB showed promising results in only one trial area, and CC provided only minor benefits. These findings highlight the necessity of site- and cultivar-specific selection for stress-alleviating treatments.

## 4. Materials and Methods

**Figure 7:**
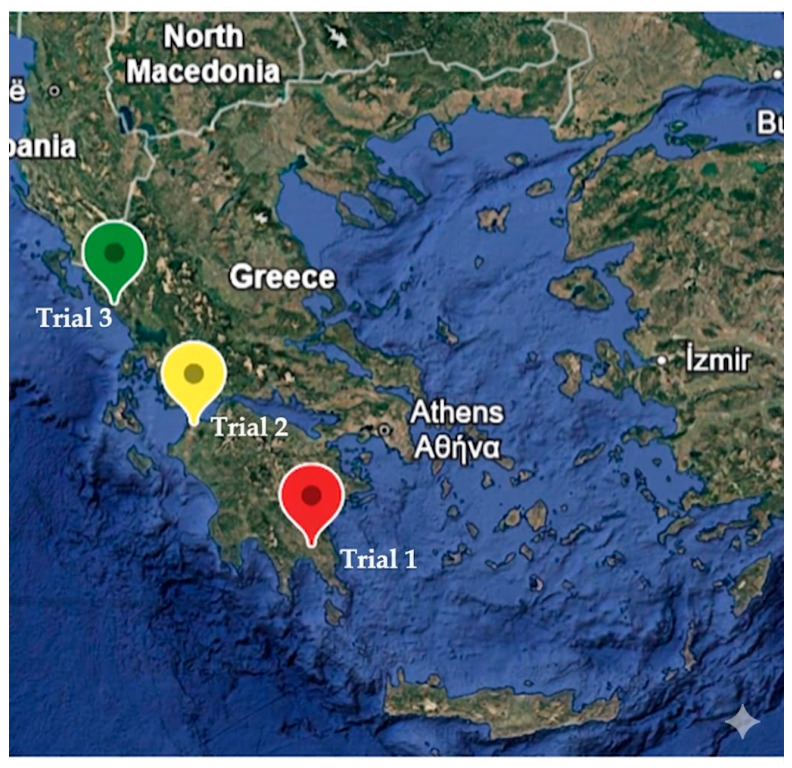
Map of Greece where the three trial locations are shown (map created by Google earth).

The study was conducted in three different sites in Greece (Figure 7). In all sites, the olive trees were cultivated under rainfed conditions.

### 4.1. Efficacy Evaluation of Various Products on “Koroneiki” Cultivar

#### 4.1.1. Trial 1

The olive grove (approximately 1.6 ha) was located in Southern Greece, in Glikovrisi village, municipality of Evrotas, in Lakonia county (36°50′01.0″ N and 22°47′04.7″ E, 22 m altitude). Twenty uniform-in-size, full bearing thirty-year-old trees of cultivar “Koroneiki”, with similar fruit load, were selected in early summer.

Three commercially available products were used in order to test the efficacy of glycine betaine, kaolin clay particles and calcium carbonate against control (sprayed only with water), i.e.,

- BlueStim (glycine betaine 95%, *w*/*w*) WP (by Lallemand Inc., Toronto, ON, Canada, distributed in Greece by Hellafarm S.A., Peania, Greece), an osmolyte, at the registered dose rate of 500 g 100 L^−1^ recommended by the supplier;
- Surround^®^ WP (kaolin—aluminum silicate 95% *w*/*w*) (Al_2_Si_2_O_5_(OH)_4_ 95%) (Tessenderlo Kerley, Inc., Phoenix, AZ, USA, distributed in Greece by Hellafarm S.A., Peania, Greece), a highly reflective processed kaolin particle film material, at the registered dose rate of 3 kg 100 L^−1^ recommended by the supplier;
- PurShade^®^ Solar Protectant (calcium carbonate 62.5% *w*/*w*) (Tessenderlo Kerley, Inc., Phoenix, AZ, USA, distributed in Greece by Hellafarm S.A., Peania, Greece), a solar protectant against ultraviolet (UV) and infrared (IR) radiation, at the registered dose rate of 2 L 100 L^−1^ recommended by the supplier.

All the products were applied 2–3 days before the anticipated heat waves in the region, using a knapsack battery driven sprayer. In all tank mixes, an adjuvant-emulsifier (Hydroil (plant based oil 100%) at 100 mL 100 L^−1^, distributed in Greece by Vioryl SA, Afidnes, Greece) was added to improve foliage coverage. Spray application was performed to the point of run off, ensuring uniform-thorough foliage coverage. Kaolin clay particles were applied twice (19 July—BBCH crop growth stage scale 74 and 27 July—BBCH crop growth stage scale 74), glycine betaine and calcium carbonate thrice (19 July, 27 July and 23 August—BBCH crop growth stage scale 76). Kaolin clay particles were not applied thrice, as the foliage coverage was enough even one month after the last spray event. Five trees were used per treatment, each one representing one replicate (thus five replicate-plots per treatment, of one tree each).

During the trial period, the mean temperature recorded in July was 30.5 °C and the mean maximum one 38.8 °C with the maximum temperature reaching 42.4 °C. In August, the mean temperature recorded was 30.9 °C and the mean maximum one 38.5 °C with the maximum temperature reaching 41.6 °C. In September, the mean temperature recorded was 30.4 °C and the mean maximum one 37.6 °C with the maximum temperature reaching 41.4 °C. A light rainfall occurred on the 26 July (8 mm) and on the period between 21 and 30 September (12.5 mm) (sources https://meteosearch.meteo.gr/, freemeteo.gr, accessed on 5 March 2026).

The harvest took place in mid-November (19 November) and at the same time, the length of at least 12 annual shoots per tree (from the periphery of each tree) was measured. Each tree was harvested separately, and the yield was recorded. Approximately 1.5 kg of free-from-leaves healthy olive fruits were randomly sampled from each plot and transferred to the laboratory for further processing. A total of five samples (1.5 kg each) per treatment were assayed.

##### Olive Oil Extraction Procedure

The maturity index of the fruits was determined in the laboratory, in a sample of 100 randomly selected olive fruits. The free-from-leaves and other debris olives were crushed using an Abencor-type olive mill (Callis S.A., Athens, Greece) and the oil percentage in the paste was determined according to Roussos et al. [2]. The oil samples were then stored in amber glass top-filled bottles at 4 ± 2 °C until analysis.

##### Olive Oil Analyses

The determination of olive oil free acidity, peroxide value, and ultraviolet absorp-tion at 232 and 270 nm (K232, K270 respectively) was conducted according to the European Official Methods of Analysis 2016/1784.

##### Determination of Total Phenols

Extraction of phenolic compounds and quantification was accomplished based on the methods described by Roussos et al. [49]. Results were expressed as mg of gallic acid (GAEs) regarding total phenol concentration, caffeic acid (CAEs) regarding total o-diphenols concentration, and catechin equivalents (CtEs) per kg of olive oil regarding total flavonoids concentration, respectively.

##### Antioxidant Capacity

The antioxidant capacity of the oils was determined based on the diphenyl-picryl hydrazyl (DPPH) and the ferric reducing antioxidant power (FRAP) assays, according to Roussos et al. [49], and expressed as μmol Trolox equivalents kg^−1^ olive oil.

#### 4.1.2. Trial 2

The olive grove (approximately 0.35 ha) was located in Western Greece, in Kagkadi village, municipality of Larissos, in Ahaia county (21°26′ 57.17″ E and 38°02′ 32.17″ N, 80 m altitude). Twelve uniform-in-size, full bearing fifteen-year-old trees of cultivar “Koroneiki”, with similar fruit load, were selected in early summer.

The products used were the same as in the first trial. The applications were conducted as follows:

- Glycine betaine was applied twice, on 22 July—BBCH crop growth stage scale 74 and 19 August—BBCH crop growth stage scale 76.
- Kaolin was applied twice, on 22 July—BBCH crop growth stage scale 74 and on 30 July—BBCH crop growth stage scale 74.
- Calcium carbonate was applied thrice, on 22 and 30 July—BBCH crop growth stage scale 74 and on 20 August—BBCH crop growth stage scale 76).

All the products were applied 2–3 days before the anticipated heat waves in the region, using a gasoline-driven sprayer. In all tank mixes, an adjuvant-emulsifier (SALDO PLUS (isodecyl alcohol ethoxylate 15% *w*/*v*) 15 SL at 30 mL 100 L^−1^, distributed in Greece by SEGE, Athens, Greece) was added to improve foliage coverage. Spray application was performed to the point of run off, ensuring uniform-thorough foliage coverage. Calcium carbonate was applied thrice due to a light rain event, which took place in mid-August and partially rinsed the product from the leaves. Three trees were used per treatment, each one representing one replicate (thus three replicate-plots per treatment, of one tree each).

During the trial period, the mean temperature recorded in July was 26.6 °C and the mean maximum one 33.3 °C with the maximum temperature reaching 39.0 °C. In August, the mean temperature recorded was 27.3 °C and the mean maximum one 34.8 °C, with the maximum temperature reaching 39.0 °C. In September, the mean temperature recorded was 23.8 °C and the mean maximum one 28.9 °C, with the maximum temperature reaching 38.0 °C. A rainfall occurred on the 8 August (12 mm), followed by rainfalls on 21–29 September (35 mm) (sources https://meteosearch.meteo.gr/, freemeteo.gr accessed on 5 March 2026).

The harvest took place on the 10 December and at the same time, the length of at least 10 annual shoots per tree (from the periphery of each tree) was measured. Each tree was harvested separately, and the yield was recorded. Approximately 1.5 kg of free-from-leaves healthy olive fruits were randomly sampled from each plot and transferred to the laboratory for further processing. A total of three samples (1.5 kg each) per treatment were assayed.

The analyses that took place were the same as those described in Trial 1.

### 4.2. Trial 3—Efficacy Evaluation of Various Products on “Lianolia Kerkyras” Cultivar

The olive grove (approximately 0.2 ha) was located in North-Western Greece, in Loutsa village, municipality of Parga, in Preveza county (39.1975° N and 20.5315° E, 287 m altitude). Sixteen uniform-in-size, full bearing thirty-year-old trees of cultivar “Lianolia Kerkyras”, with similar fruit load, were selected in early summer.

The products used were the same as in the first trial. The applications were conducted as follows:

- Glycine betaine was applied twice, on 23 July—BBCH crop growth stage scale 79 and 21 August—BBCH crop growth stage scale 79.
- Kaolin was applied thrice, on 23 July and 13 and 29 August—BBCH crop growth stage scale 78–79.
- Calcium carbonate was applied thrice, on 23 July and 13 and 29 August—BBCH crop growth stage scale 78–79).

All the products were applied 2–3 days before the anticipated heat waves in the region, using a gasoline-driven sprayer. In all tank mixes, an adjuvant-emulsifier (SALDO PLUS (isodecyl alcohol ethoxylate 15% *w*/*v*) 15 SL at 30 mL 100 L^−1^, distributed in Greece by SEGE, Greece) was added to improve foliage coverage. Spray application was performed to the point of run off, ensuring uniform-thorough foliage coverage. Kaolin and calcium carbonate were applied three times due to persisting rain events in August, which partially rinsed the products from the leaves. Four trees were used per treatment, each one representing one replicate (thus four replicate-plots per treatment, of one tree each).

During the trial period, the mean temperature recorded in July was 26.7 °C and the mean maximum one 31.2 °C, with the maximum temperature reaching 35.9 °C. In August, the mean temperature recorded was 27.5 °C and the mean maximum one 32.8 °C, with the maximum temperature reaching 37.8 °C. In September, the mean temperature recorded was 23.8 °C and the mean maximum one 28.6 °C, with the maximum temperature reaching 34.1 °C. Multiple events of rainfall occurred between the period of 5–13 August (total of 40 mm), and a prolonged rainfall event during the period of 17–29 September (122 mm).

The harvest took place on the 15 November. Each tree was harvested separately, and the yield was recorded. Approximately 1.5 kg of free-from-leaves healthy olive fruits were randomly sampled from each plot and transferred to the laboratory for further processing. A total of four samples (1.5 kg each) per treatment were assayed.

The analyses of olive oil that took place were the same as those described in Trial 1. Furthermore, the mean fruit weight, length and diameter were assayed using a digital caliper (Parkside, Lidl, Neckarsulm, Germany).

Olive oil functional properties were further tested, by measuring the α-tocopherol and individual phenolic compound concentration as well as the fatty acid profile and squalene concentration.

#### 4.2.1. α-Tocopherol Determination

The concentration of α-tocopherol was evaluated based on the protocol by Roussos et al. [2]. Briefly, 100 μL of olive oil was diluted in 900 μL of HPLC-grade hexane and homogenized via vortexing. Analysis was performed using a normal-phase HPLC system equipped with a Lichrosorb Si 60 column (250 × 4.6 mm, 5 μm; Merck KGaA, Darmstadt, Germany) maintained at 20 °C. The mobile phase consisted of an isocratic mixture of hexane and 2-propanol (99:1 *v*/*v*) at a flow rate of 0.8 mL min^−1^. Detection was carried out using a fluorescence detector (HP 1046A, Agilent Technologies, Santa Clara, CA, USA) with excitation and emission wavelengths set at 290 nm and 325 nm, respectively. α-tocopherol was identified and quantified through a five-point calibration curve using an authentic standard (Sigma-Aldrich, St. Louis, MO, USA).

#### 4.2.2. Individual Phenolic Compounds Determination

Individual phenolic compounds were detected in the same phenolic extract produced during the extraction of total phenolic compounds (as described in Section 4.1.1) according to Roussos et al. [2]. Briefly, a sample of 40 μL was injected in a Luna LC column 250 × 4.6 mm 5 μm C18 (2) 100 A (Phenomenex, 411 Madrid Avenue, Torrance, CA, USA) (coupled with a guard column Gemini C18 4 × 3.0 mm) (Phenomenex, 411 Madrid Avenue, Torrance, CA, USA), thermostated at 20 °C. The various phenolic compounds were detected using a high-performance liquid chromatography (HPLC), Shimadzu Nexera X2 (Shimadzu Europa GmbH, Albert-Hahn-Str. 6-10, Duisburg, Germany), equipped with a diode array detector scanning the eluted compounds at 260 nm, 280 nm, 325 nm, and 360 nm. The elution of the compounds of interest was accomplished by a gradient program of two mobile phases, i.e., (A) a mixture of methanol:acetonitrile at a ratio 1:1 and (B) a solution of 1% *v*/*v* acetic acid in water. Chromatographic separation was performed at a constant flow rate of 1 mL min^−1^. The gradient elution began with 10% solvent A, maintained for the initial 5 min. Subsequently, the proportion of A was increased to 20% (from 10 to 35 min), 25% (45 to 55 min), 30% (at 70 min), and 40% (at 100 min), reaching 70% during the final 106–123 min interval. Identification of eleven (11) phenolic constituents (hydroxytyrosol, tyrosol, vannilic acid, caffeic acid, vannilin, p-coumaric acid, ferulic acid, oleacein, oleocanthal, luteolin, and apigenin) was achieved by comparing retention times and spectral data with commercial standards. These standards were sourced from Sigma-Aldrich (St. Louis, MO, USA), with final concentrations reported in mg kg^−1^ of olive oil.

#### 4.2.3. Fatty Acid Composition of Total Lipids and Squalene Determination in Olive Oil

The profile of fatty acids of total lipids was analyzed following the methodology described by Roussos et al. [2] as their responding methyl esters (FAMEs). For the derivatization, 0.5 g of the oil sample was dissolved in 5 mL GC-grade hexane, followed by vigorous agitation and the addition of 0.5 mL of methanolic KOH (0.2 N). The samples were vortexed for 30 sec and left at room temperature for 30 min. The upper phase was carefully removed and used for the analysis. Chromatographic analysis was conducted using a Shimadzu GC-2030 system (Duisburg, Germany) featuring an AOC-20i+s autosampler and a flame ionization detector (FID). Separation was achieved on a Supelco SP^®^-2340 capillary column (60 m × 0.32 mm × 0.2 μm). With a split ratio of 80:1 and both injector and detector temperatures maintained at 249 °C, 1 µL of sample was injected. The oven program started at 150 °C (18 min hold), rose to 175 °C at 2 °C min^−1^ (5 min hold), and finally reached 200 °C at 5 °C min^−1^ (22 min hold). The fatty acids identified included palmitic (C16:0), palmitoleic (C16:1), stearic (C18:0), oleic (C18:1), linoleic (C18:2), linolenic (C18:3), arachidic (C20:0), and gadoleic (C20:1). FAMES were identified using the 37 component FAME mix of Supelco (Merck Group, St. Louis, MO, USA). Squalene was also determined under the same conditions (the area of its peak was not taken into account for the calculation of the total area of FAMES). Squalene concentration was quantified via a five-point calibration curve using analytical standards (Sigma-Aldrich, St. Louis, MO, USA) and reported as mg 100 g^−1^ of oil.

### 4.3. Statistical Analysis

All the trials followed the completely randomized design with three–five replicates of one tree (depending on the trial), in each orchard, i.e., three–five trees per treatment. The raw data from each olive grove were separately analyzed as a one-way ANOVA. Significant differences among treatments were determined based on the Duncan’s multiple range test, at α = 0.05 after checking the normal distribution of the raw data using standard skewness, standard kurtosis, and the homogeneity of variances. When necessary, the suitable transformation of the raw data was performed in order to get a normal distribution. Discriminant analysis of raw data took place in order to investigate possible differentiation among treatments based on their effects on both shoots, fruit and oil characteristics (according to each trial). Hierarchical cluster analysis (Ward method) of all the raw data per trial was performed to produce a dense, descriptive information on the effects of the various treatments. Constellation plots were also constructed to graphically present possible similarities of the various treatments based on the measured variables. The statistical software JMP 13.0 (SAS Institute, Cary, NC, USA) and Statgraphics Centurion XV (Statgraphics Technologies, Inc., The Plains, VA, USA) were used for the aforementioned analyses.

## 5. Conclusions

Summer stress poses a major threat to olive productivity, but targeted foliar applications of glycine betaine and kaolin can effectively safeguard yield and oil production. Kaolin outperformed the other treatments, although GB showed promise in one of the three trials. In contrast, CC exhibited limited efficacy. While the impacts on oil quality were modest, GB and K differentially influenced phenolic content and antioxidant capacity under stress conditions. These findings demonstrate that cultivar- and site-specific foliar treatments offer a sustainable strategy for maintaining olive productivity and preserving functional oil quality in the face of climate change.

## Figures and Tables

**Table 1:** Effects of the various treatments on “Koroneiki” olive fruit yield, olive oil percentage, oil produced per tree, fruit maturity index and mean annual shoot length at harvest.

Treatments	Yield (kg Tree^−1^)	Oil Percentage per Fruit	Oil per Tree (kg)	Maturity Index	Shoot Length (cm)
	“Koroneiki” cultivar in trial site 1				
C	19.16 ± 2.3 bc	18.11 ± 1.8 a	3.48 ± 0.6 bc	3.17 ± 0.8 a	4.54 ± 0.7 a
CC	14.76 ± 2.5 c	19.71 ± 2.0 a	2.93 ± 0.8 c	3.76 ± 0.2 a	5.20 ± 1.5 a
GB	24.37 ± 4.3 a	19.34 ± 0.8 a	4.69 ± 0.7 a	3.51 ± 1.0 a	6.47 ± 1.5 a
K	22.53 ± 4.0 ab	19.46 ± 0.5 a	4.38 ± 0.8 ab	3.61 ± 0.4 a	4.97 ± 0.8 a
	“Koroneiki” cultivar in trial site 2				
C	68.0 ± 2.0 b	14.4 ± 1.7 a	9.81 ± 1.4 c	1.8 ± 0.6 a	5.75 ± 0.3 a
CC	84.67 ± 9.5 ab	15.5 ± 0.1 a	12.99 ± 0.3 ab	1.7 ± 0.4 a	5.84 ± 1.1 a
GB	90.0 ± 8.7 a	12.9 ± 0.3 a	11.59 ± 0.9 bc	1.7 ± 0.4 a	5.71 ± 0.6 a
K	97.3 ± 7.6 a	15.4 ± 1.6 a	14.99 ± 1.1 a	1.5 ± 0.1 a	5.60 ± 0.6 a

Means ± standard deviation within the same column, separately per trial site, followed by the same letter, do not differ significantly based on Duncan’s multiple range test at α = 0.05. Abbreviations: C, control, CC, calcium carbonate, GB, glycine betaine, K, kaolin.

**Table 2:** Effects of the various treatments on “Lianolia Kerkyras” olive fruit yield, olive oil percentage, oil produced per tree, fruit maturity index, and mean fruit weight, diameter and length.

Treatment	Yield (kg Tree^−1^)	Oil Percentage per Fruit	Oil per Tree (kg)	Maturity Index	Fruit Weight (g)	Fruit Diameter (mm)	Fruit Length (mm)
C	109.8 ± 13.4 b	13.2 ± 2.38 a	14.42 ± 2.93 b	1.34 ± 0.23 a	4.81 ± 0.35 a	9.65 ± 0.44 a	16.13 ± 1.19 a
CC	97.95 ± 18.6 b	13.2 ± 1.48 a	12.87 ± 2.59 b	1.71 ± 0.86 a	5.12 ± 0.39 a	9.86 ± 0.47 a	16.25 ± 0.58 a
GB	95.47 ± 13.9 b	12.6 ± 1.61 a	12.04 ± 2.43 b	1.62 ± 0.42 a	4.50 ± 0.56 a	9.92 ± 0.71 a	16.00 ± 0.98 a
K	142.7 ± 31.8 a	13.8 ± 1.62 a	19.42 ± 3.84 a	1.69 ± 0.62 a	5.03 ± 0.69 a	10.15 ± 0.81 a	17.13 ± 0.77 a

Means ± standard deviation within the same column, followed by the same letter, do not differ significantly based on Duncan’s multiple range test at α = 0.05. Abbreviations: C, control, CC, calcium carbonate, GB, glycine betaine, K, kaolin.

**Table 3:** Effects of the various treatments on oil free acidity, peroxide index, K232 and K270 in all trial sites.

Treatments	Free Acidity (g Oleic Acid 100 g^−1^)	Peroxides (meq O_2_ kg^−1^)	K232	K270
	“Koroneiki” cultivar in trial site 1			
C	0.28 ± 0.05 a	17.7 ± 2.2 a	2.29 ± 0.21 a	0.194 ± 0.029 a
CC	0.22 ± 0.03 ab	17.6 ± 2.5 a	2.34 ± 0.16 a	0.212 ± 0.008 a
GB	0.28 ± 0.00 a	17.1 ± 3.0 a	1.99 ± 0.89 a	0.194 ± 0.011 a
K	0.21 ± 0.07 b	18.8 ± 1.1 a	2.27 ± 0.16 a	0.186 ± 0.032 a
	“Koroneiki” cultivar in trial site 2			
C	0.19 ± 0.04 a	16.1 ± 2.0 a	1.79 ± 0.36 a	0.164 ± 0.024 a
CC	0.16 ± 0.04 a	16.2 ± 1.3 a	2.08 ± 0.10 a	0.162 ± 0.005 a
GB	0.19 ± 0.04 a	18.9 ± 2.0 a	1.98 ± 0.23 a	0.162 ± 0.017 a
K	0.19 ± 0.04 a	17.4 ± 3.3 a	2.12 ± 0.08 a	0.180 ± 0.009 a
	“Lianolia Kerkyras” cultivar in trial site 3			
C	0.26 ± 0.07 a	13.8 ± 2.5 a	1.90 ± 0.21 a	0.196 ± 0.01 a
CC	0.31 ± 0.08 a	12.5 ± 2.9 a	1.94 ± 0.14 a	0.211 ± 0.00 a
GB	0.37 ± 0.09 a	11.3 ± 2.5 a	1.77 ± 0.21 a	0.185 ± 0.04 a
K	0.30 ± 0.18 a	11.3 ± 2.5 a	1.86 ± 0.12 a	0.211 ± 0.01 a

Means ± standard deviation within the same column, separately per trial, followed by the same letter, do not differ significantly based on Duncan’s multiple range test at α = 0.05. Abbreviations: C, control, CC, calcium carbonate, GB, glycine betaine, K, kaolin.

**Table 4:** Effects of the various treatments on oil total phenols, o-diphenols, and flavonoid concentration and antioxidant capacity based on FRAP and DPPH assays (in μmol Trolox equivalents kg^−1^) in all trial sites.

Treatments	Total Phenols (mg GAE kg^−1^)	Total o-Diphenols (mg CAE kg^−1^)	Total Flavonoids (mg CtE kg^−1^)	FRAP	DPPH
	“Koroneiki” cultivar in trial site 1				
C	299.39 ± 82.7 a	240.74 ± 91.3 a	339.45 ± 104.1 a	1407.5 ± 256.7 a	908.7 ± 287.8 a
CC	192.25 ± 27.1 b	487.73 ± 175.8 a	279.06 ± 76.8 a	1276.7 ± 296.2 a	714.9 ± 184.6 a
GB	317.89 ± 79.9 a	414.00 ± 91.5 a	286.19 ± 124.7 a	1464.6 ± 472.4 a	837.6 ± 328.8 a
K	287.08 ± 40.7 ab	290.41 ± 89.2 a	292.65 ± 98.2 a	1192.5 ± 192.7 a	744.3 ± 245.9 a
	“Koroneiki” cultivar in trial site 2				
C	102.7 ± 16.6 a	28.16 ± 4.6 a	141.20 ± 26.5 a	497.7 ± 203.5 a	314.6 ± 11.8 ab
CC	66.13 ± 3.2 bc	32.00 ± 3.4 a	120.40 ± 19.3 a	378.3 ± 83.6 a	131.8 ± 10.0 c
GB	46.24 ± 5.3 c	25.50 ± 0.7 a	53.92 ± 20.7 b	327.9 ± 109.3 a	238.4 ± 35.9 b
K	77.97 ± 14.1 ab	27.78 ± 4.6 a	155.49 ± 1.4 a	421.9 ± 257.3 a	356.6 ± 47.0 a
	“Lianolia Kerkyras” cultivar in trial site 3				
C	513.4 ± 46.3 ab	46.88 ± 15.53 a	240.0 ± 46.3 a	1478.0 ± 384.7 a	1831.5 ± 208.5 a
CC	608.4 ± 38.1 a	38.24 ± 2.68 a	266.5 ± 55.2 a	1543.9 ± 205.4 a	1452.8 ± 285.0 a
GB	516.7 ± 83.8 ab	34.89 ± 5.25 a	209.9 ± 55.5 a	1492.3 ± 223.0 a	1546.7 ± 177.8 a
K	418.5 ± 58.0 b	16.48 ± 9.87 b	265.5 ± 44.4 a	1541.9 ± 257.6 a	1680.3 ± 115.5 a

Means ± standard deviation within the same column, separately per trial, followed by the same letter, do not differ significantly based on Duncan’s multiple range test at α = 0.05. Abbreviations: C, control, CC, calcium carbonate, GB, glycine betaine, K, kaolin, GAE, gallic acid equivalent, CAE, caffeic acid equivalent, CtE, catechin equivalent.

**Table 5:** Effects of the various treatments on the concentration of individual phenolic compounds and α-tocopherol detected in “Lianolia Kerkyras” olive oils (in mg kg^−1^).

Treatment	Hydroxytyrosol	Tyrosol	Vanillic Acid	Caffeic Acid	Vanillin	p-Coumaric Acid	Ferulic Acid	Oleacein	Oleocanthal	Luteolin	Apigenin	α-Tocopherol
C	11.86 ± 6.08 a	12.72 ± 4.33 a	0.98 ± 0.37 a	0.023 ± 0.01 a	0.47 ± 0.04 a	0.31 ± 0.14 a	0.053 ± 0.017 a	229.2 ± 110.3 a	413.7 ± 74.1 a	22.07 ± 3.05 a	0.60 ± 0.42 a	32.15 ± 17.44 bc
CC	12.41 ± 2.41 a	12.82 ± 2.95 a	1.12 ± 0.14 a	0.028 ± 0.000 a	0.53 ± 0.07 a	0.46 ± 0.23 a	0.055 ± 0.013 a	288.7 ± 15.5 a	533.9 ± 52.3 a	20.65 ± 9.79 a	0.57 ± 0.29 a	49.44 ± 13.99 ab
GB	9.73 ± 1.15 a	10.65 ± 1.86 a	1.15 ± 0.26 a	0.025 ± 0.001 a	0.68 ± 0.14 a	0.59 ± 0.12 a	0.052 ± 0.005 a	272.8 ± 67.0 a	441.5 ± 90.0 a	21.13 ± 6.32 a	0.59 ± 0.13 a	65.11 ± 12.20 a
K	11.64 ± 0.92 a	13.28 ± 2.46 a	1.07 ± 0.26 a	0.028 ± 0.010 a	0.54 ± 0.11 a	0.47 ± 0.20 a	0.065 ± 0.006 a	252.3 ± 23.9 a	504.1 ± 167.7 a	26.46 ± 7.94 a	0.73 ± 0.22 a	28.41 ± 0.17 c

Means ± standard deviation within the same column followed by the same letter do not differ significantly based on Duncan’s multiple range test at α = 0.05. Abbreviations: C, control, CC, calcium carbonate, GB, glycine betaine, K, kaolin.

**Table 6:** Fatty acid composition of total lipids (as the responding FAMEs %) in “Lianolia Kerkyras” olive oil under the effects of the various treatments.

Treatment	C16:0	C16:1	C18:0	C18:1	C18:2	C20:0	C18:3	C20:1
C	16.74 ± 0.13 a	1.06 ± 0.25 a	1.96 ± 0.09 a	70.37 ±a	8.79 ± 0.23 a	0.33 ± 0.01 a	0.71 ± 0.16 a	0.18 ± 0.09 a
CC	16.77 ± 0.30 a	1.07 ± 0.14 a	1.94 ± 0.20 a	69.80 ±a	9.18 ± 0.52 a	0.21 ± 0.09 b	0.77 ± 0.13 a	0.29 ± 0.07 a
GB	16.61 ± 0.67 a	1.14 ± 0.17 a	1.85 ± 0.17 a	69.83 ±a	9.22 ± 0.36 a	0.13 ± 0.18 b	0.75 ± 0.12 a	0.44 ± 0.20 a
K	16.58 ± 0.15 a	1.12 ± 0.17 a	1.84 ± 0.17 a	69.77 ±a	9.40 ± 0.65 a	0.16 ± 0.16 b	0.69 ± 0.10 a	0.43 ± 0.26 a

Means ± standard deviation within the same column followed by the same letter do not differ significantly based on Duncan’s multiple range test at α = 0.05. Abbreviations: C, control, CC, calcium carbonate, GB, glycine betaine, K, kaolin.

**Table 7:** Fatty acid composition of total lipids (as the responding FAMEs %) and squalene concentration in “Lianolia Kerkyras” olive oil under the effects of the various treatments.

Treatment	SFAs	MUFAs	PUFAs	UFAs	MUFAs/PUFAs	SFAs/UFAs	C18:1/C18:2	Squalene (mg 100 g^−1^)
C	18.87 ± 0.22 a	71.61 ± 0.32 a	9.51 ± 0.32 a	81.12 ± 0.22 a	7.53 ± 0.29 a	0.23 ± 0.00 a	0.027 ± 0.001 a	179.3 ± 27.7 a
CC	18.87 ± 0.59 a	71.16 ± 0.64 a	9.95 ± 0.52 a	81.12 ± 0.59 a	7.16 ± 0.41 a	0.23 ± 0.01 a	0.028 ± 0.003 a	165.7 ± 23.1 ab
GB	18.60 ± 0.67 a	71.41 ± 1.00 a	9.98 ± 0.35 a	81.40 ± 0.67 a	7.16 ± 0.35 a	0.23 ± 0.01 a	0.027 ± 0.003 a	150.9 ± 37.6 ab
K	18.57 ± 0.21 a	71.33 ± 0.63 a	10.09 ± 0.75 a	81.42 ± 0.20 a	7.10 ± 0.57 a	0.23 ± 0.00 a	0.027 ± 0.003 a	121.5 ± 19.6 b

Means ± standard deviation within the same column, separately per trial, followed by the same letter, do not differ significantly based on Duncan’s multiple range test at α = 0.05. Abbreviations: C, control, CC, calcium carbonate, GB, glycine betaine, K, kaolin.

## Data Availability

Data are available upon request to the corresponding author.
